# Defining a Time Window for Neuroprotection and Glia Modulation by Caffeine After Neonatal Hypoxia-Ischaemia

**DOI:** 10.1007/s12035-020-01867-9

**Published:** 2020-01-23

**Authors:** Elena Di Martino, Erica Bocchetta, Shunichiro Tsuji, Takeo Mukai, Robert A. Harris, Klas Blomgren, Ulrika Ådén

**Affiliations:** 1 Department of Women’s and Children’s Health, Karolinska Institutet, BioClinicum J9:30 Visionsgatan 4, 17176 Stockholm, Sweden; 2 Department of Life Science, University of Trieste, Trieste, 34123 Italy; 3 Department of Obstetrics and Gynaecology, Shiga University of Medical Science, Shiga, 522-8522 Japan; 4 Department of Clinical Neuroscience, Karolinska Institutet, Centre for Molecular Medicine, Karolinska University Hospital, 17176 Stockholm, Sweden; 5 Paediatric Oncology, Karolinska University Hospital, 17176 Stockholm, Sweden; 6 Neonatology, Karolinska University Hospital, 17176 Stockholm, Sweden

**Keywords:** Hypoxia-ischemia, Caffeine, Time-window, Neuroprotection, Immunomodulation

## Abstract

Hypoxic-ischemic (HI) brain injury remains an important cause of brain damage in neonates with potential life-long consequences. Caffeine, which is a competitive inhibitor of adenosine receptors, is commonly used as treatment for preterm apnoea in clinical settings. In the current study, we investigated the effects of caffeine given at 0 h, 6 h, 12 h or 24 h after HI in P10 mouse pups. Open field and rotarod behavioural tests were performed 2 weeks after injury, and brain morphology was then evaluated. Gene expression and immunohistological analyses were assessed in mice 1- and 5-day post-HI. A single dose of caffeine directly after HI resulted in a reduction of the lesion in the grey and white matter, judged by immunostaining of MAP2 and MBP, respectively, compared to PBS-treated controls. In addition, the number of amoeboid microglia and apoptotic cells, the area covered by astrogliosis, and the expression of pro-inflammatory cytokines were significantly decreased. Behavioural assessment after 2 weeks showed increased open-field activity after HI, and this was normalised if caffeine was administered immediately after the injury. Later administrations of caffeine did not change the outcomes when compared to the vehicle group. In conclusion, caffeine only yielded neuroprotection and immunomodulation in a neonatal model of brain hypoxia ischaemia if administered immediately after injury.

**Electronic supplementary material:**

The online version of this article (10.1007/s12035-020-01867-9) contains supplementary material, which is available to authorized users.

## Introduction

Perinatal hypoxia ischaemia (HI) remains a main cause of morbidity and mortality in neonates, the latter accounting for 45% of the 5.9 million child deaths that occurred in 2015 globally, with the highest incidence in low-income countries [1]. HI, during or after birth, may affect the brain and lead to serious neurological consequences, including cerebral palsy, cognitive deficits, motor impairment and epilepsy [2].

Mild hypothermia is currently the only treatment that has proven to improve survival without disability in neonates with moderate (but not severe) HI [3]. However, this neuroprotective strategy demands intensive care support, and potential side effects have to be considered, especially in low-income settings [4]. Development of alternative treatment strategies is ongoing.

Caffeine is the most commonly used psychoactive drug worldwide. It is a non-specific adenosine receptor (AR) antagonist and binds to some extent to three of the ARs (named A1, A2a, A2b) with a preference for A1R and A2aR under normal physiological conditions [5]. In the brain, these receptors are expressed in the pre- and post-synaptic site of neurons, respectively, but are also present in other cells, such as astrocytes, oligodendrocytes and microglia [6]. In addition, the A2aR is implicated in neuroinflammation, and its expression in microglial cells is increased after brain injury [7].

Caffeine has been routinely used for decades in the clinic for apnoeic children [8], and a randomised controlled study to treat apnoea showed reduced incidences of cerebral palsy and cognitive delay in caffeine-treated patients when compared with the placebo group [9]. Pre-clinical studies have also reported a neuroprotective effect of caffeine in experimental models of neonatal HI, resulting in behavioural and functional recovery [10–13]. Previous works from us and others have demonstrated that caffeine reduced a mild lesion in mouse models and/or ameliorated behavioural performances when given before [13] or immediately after injury [10, 12], or for multiple days following HI [11]. From a clinical and practical point of view, it is important to know if the single-dose therapy is effective at 6–24 h after injury and at different stages of injury severity. Such data are not available to our knowledge. In addition, the effects of caffeine on cellular injury and repair in neonatal models have not been reported.

We therefore aimed to study the time window of the neuroprotective effect of one dose of caffeine given after neonatal hypoxic ischemia using morphological, behavioural and neuroinflammatory outcomes.

## Materials and Methods

All experiments were approved by the regional ethics committee, Stockholms norra djurförsöksetiska nämnd, in accordance with local institutional guidelines and the Directive N249/13. All methods were carried out in accordance with relevant guidelines and regulations.

### Hypoxic-Ischemic Brain Injury

Wild type C57/bl6 specific pathogen-free mice were bred in house. Dams and pups had free access to pelleted food and were housed in open cages with standard enrichment and daily monitoring in accordance with local institutional guidelines. On postnatal day 10 (P10), a modified version of the Vannucci model [14] was performed on pups of both sexes deriving from more than 10 l. Briefly, unilateral electrocoagulation (8 W) of the right carotid artery was conducted via a midline neck incision under isoflurane anaesthesia and local bupivacaine infiltration to minimise pain and distress. Pups were returned to the dam for 1 h for feeding purposes and then subjected to hypoxia (10% O_2_ in 90% N_2_ at 36 °C). There was no mortality, severe illness or need for early euthanasia. During sham-operation, the carotid artery was visualised and isolated but not electrocoagulated.

### Treatment

Immediately after the 60 min of hypoxia, body temperature was assessed via axillary measurement and the mice were randomised to different groups.

A single dose of 5 mg/kg caffeine or vehicle (sterile PBS in a comparable volume) was administered intraperitoneally (i.p.) to injured and sham pups either directly after HI (0 h), or at 6, 12, and 24 h (h) post injury.

The dose of caffeine was chosen to reflect clinically relevant serum concentrations of caffeine when administered to neonates for the prevention of apnoea [15–17].

### Behavioural Tests

Behavioural assessments were performed 2 weeks after HI in all groups (sham, caffeine- or PBS-treated animals), starting at P24, for three consecutive days by an investigator blinded to the lesion and treatment.

#### Open-Field Test

Mice were tested in groups of four, randomising sex and experimental condition, and placed in four square arenas (50 cm per side). Mice were handled with care to assure calmness, and their locomotion was investigated for 30 min in the dark, using an infrared light (BIOBSERVE GmbH Software, St. Augustin, Germany).

#### Rotarod Test

Mice were subjected to a rotarod test (Ugo Basile) on the last day of behavioural evaluations. They were placed on a rotating cylinder for 5 min with increasing speed (4 to 40 rpm), and the time each mouse managed to stay was recorded. The test was performed five consecutive times, and the average latency until they fell off the cylinder was calculated.

### Tissue Preparation and Cutting

The brains were collected after the end of behavioural tests (P27) for evaluation of the atrophy, or at earlier time points for histological (P15) and gene expression (P11) analyses. Animals were sacrificed by injection of 50 mg/kg sodium pentobarbital (APL, Stockholm) i.p. and cardially perfused with PBS (Life Technologies) to remove intravascular blood cells. The brains were extracted, snap-frozen in dry ice and stored at − 80 °C until sectioning. In accordance with previous studies [18], 10-μm sections were collected at three levels of each brain, corresponding to bregma 1.05 mm, − 1.76 mm and − 2.78 mm of the adult mouse brain [19], using a Leica cryostat.

### Evaluation of Brain Injury

Tissue sections were fixed for 30 min in 4% paraformaldehyde and then subjected to Nissl staining. In brief, slides were dehydrated in increasing concentrations of ethanol and xylene, incubated for 10 min in a 0.1% cresyl violet solution containing glacial acetic acid, and differentiated in 95% ethanol.

Brain injury in different regions was blindly evaluated by two independent investigators using a semi-quantitative neuropathological scoring system as described earlier [20]. Briefly, four different brain regions were scored depending on the size of the damage for a total of 0–22 points. The confluency of the injury in the cortex was rated 0–4, while hippocampus, thalamus and striatum were rated a total of 0–6 each considering the hypotrophy (0–3) and the visible infarction (0–3) for each of the areas.

### Immunohistochemistry and Immunofluorescence

Endogenous peroxidases were blocked by 0.3% H_2_O_2_ in 3% Normal Horse Serum for 10 min after 30 min fixation with 4% paraformaldehyde. VECTOR® M.O.M.™ Immunodetection Kit was used according to the manufacturer’s specifications for mouse anti microtubule-associated protein 2 (MAP2, 1:1000, Sigma) and mouse anti myelin basic protein (MBP, 1:1000, Covance) staining. For astrocytes labelling, tissue slides were incubated overnight with rabbit anti glial fibrillary acidic protein (GFAP, 1:500, Millipore) in blocking solution containing 3% donkey serum and 0.1% triton, after an initial fixation and 0.3% H_2_O_2_ peroxidase blockage. Biotinytaled donkey anti rabbit secondary antibody was applied for 1 h before incubation with VECTASTAIN® Elite ABC-Peroxidase Kit for GFAP. The enzymatic colouration of immunoreactivity for MAP2, MBP and GFAP was performed by 2-min immersion in 3,3′-diaminobenzidine (DAB, DAKO) for all slides.

To evaluate microglia morphology, tissue sections were fixed and incubated overnight with rabbit anti ionized calcium-binding adapter molecule 1 (Iba1, 1:1000, Wako) primary antibody in 3% donkey serum and 0.1% triton blocking solution. The following day the sections were then incubated with Alexa-488 donkey anti rabbit (1:1000, Life Technologies) and Hoechst (1:1000, Thermofisher) for 2 h for fluorescent immunolabeling, and mounted with ProLong Gold antifade mounting media (Life Technologies).

Neuronal apoptosis was evaluated by terminal deoxynucleotidyl transferase-mediated dUTP nick end-labeling (TUNEL) following the manufacturer’s instructions (Invitrogen).

### Tissue Loss Measurement and Quantifications

The infarction area, as judged by MAP2 unstained tissue and the glial scar evaluated by GFAP^+^ cells, were manually delineated by an investigator blinded to the lesion and treatment (Supplementary Fig. S1). The brains from sham-operated animals were also stained as negative controls and no injury or glial scar were observed as expected.

The percentage of the area covered by the injury or glial scar (%A) was determined by subtracting the areas of interest (AOI) from the total area of the ipsilateral (I) hemisphere in ratio with the area of the contralateral (C) hemisphere: [%A = 100 − [(I − AOI)/C] × 100].

To calculate the density (*D*) of amoeboid microglia or TUNEL^+^ cells, quantification analyses were performed counting the number of positive cells (*C*) in the AOI considering the thickness of the section (*T*): [*D* = *C*/(AOI × T)].

The body size of Iba1^+^ cells was analysed measuring the area of the soma of at least 20 amoeboid cells per cortex using ImageJ software, in at least three images randomly acquired in the AOI with a × 40 oil objective.

Lastly, MBP density was calculated by measuring the │mean grey intensity-background noise│ with ImageJ software (where dark and light tones correspond to low and high values respectively), in three images per AOI acquired with a × 40 oil objective.

All quantification and densitometric analyses were performed by an investigator blinded to the lesion and treatment.

Unless otherwise specified, the analyses were performed using a Zeiss AX10 bright-field microscope and Stereo-Investigator Software (MicroBrightField Inc). For TUNEL staining, images were acquired with a Zeiss LSM700 confocal microscope, and a blinded offline analysis was performed using Zen Black Software in images.

### RT-qPCR Analysis

Real-time quantitative polymerase chain reaction (RT-qPCR) was performed on cortex tissue dissected from the ipsilateral hemispheres of mice belonging to each lesion/treatment group. Messenger ribonucleic acid (mRNA) extraction was performed using the RNAeasyMini kit (Qiagen) according to the manufacturer’s instructions. cDNA was synthetized using iScript™ cDNA Synthesis Kit (BioRad). RT-qPCR was run with Power SYBR Green PCR Master Mix (Applied Biosystems) in a Step-One-Plus Real-Time PCR machine (Applied Biosystems). For all primers (Supplementary Table 1), we used an initial cycle of 10 min at 95 °C, followed by repeated cycles of 95 and 60 °C (40 in total). Melting curves, starting at 95 °C, were acquired to ensure proper primer functionality and detect any primer-dimer formation. Each gene was normalised to the average of the housekeeping genes *B-actin* and *Rpl13a*, and the expression was calculated using the ΔΔCT method.

### Statistical Analysis

The Kolmogorov-Smirnov test was used to check the normality of the data. All data were normally distributed, and the tests applied were the following: unpaired *t* test or one-way ANOVA with Bonferroni’s multiple comparison test were used for the neuropathological scores, MAP2, GFAP and TUNEL analyses, and rotarod behavioural test; two-way ANOVA with Sidak’s multiple comparison test was used for MBP and Iba1 analyses; two-way ANOVA with Dunnet’s multiple comparison test for repeated measurements was used for open-field behavioural tests. Data from the RT-qPCR analyses were additionally transformed using the *y* = log(2) function prior to the statistics. GraphPad Prism 8 software was used for all statistical analyses. Data are presented as mean ± SEM and *p* < 0.05 was considered statistically significant.

## Results

### A Single Dose of Caffeine Offers Neuroprotection Only if Administered Acutely After the Injury

**Fig. 1:**
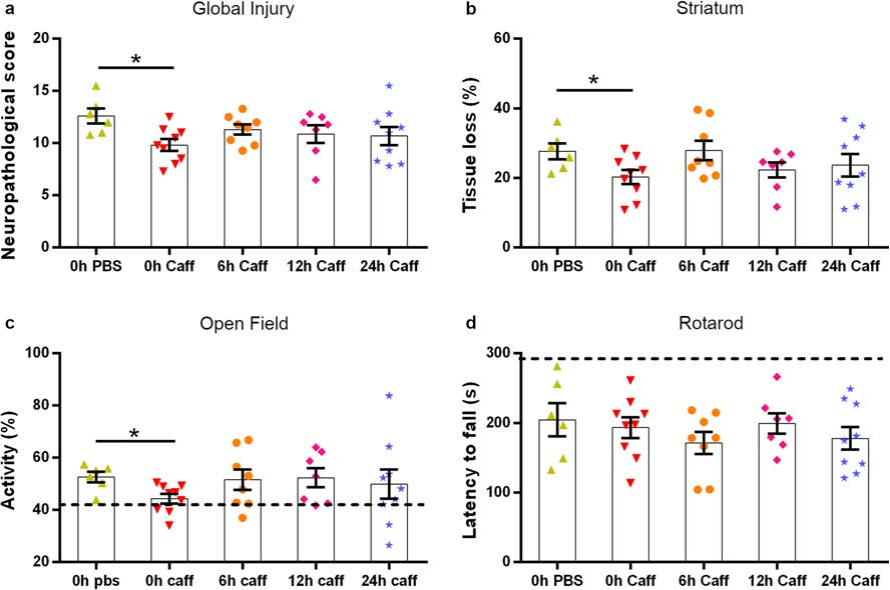
Long-term evaluation of caffeine treatment (P27). Mice were subjected to HI and received PBS (*n* = 8) or caffeine (*n* = 10) acutely (0 h) or at either 6 h (*n* = 6), 12 h (*n* = 6) and 24 h (*n* = 7) after injury. Neuropathological score in sections stained with cresyl violet (**a**), tissue loss calculated by MAP2-stained tissue (**b**), third day activity in the open field test (**c**) and rotarod (**d**) behavioural performances 2 weeks after HI. The dashed-lines in figures (**c**) and (**d**) represent the performance of sham animals as an internal control. Data are presented as mean ± SEM with *p < 0.05 (two-way ANOVA with Dunnet’s multiple comparison test for repeated measurements for open-field test and one-way ANOVA with Bonferroni’s multiple comparison test for the other graphs)

To assess the potential therapeutic effect of caffeine, P10 mice underwent HI brain injury and received PBS or 5 mg/kg caffeine immediately after surgery (0 h), 6 h, 12 h, or 24 h later in a randomised manner (Experimental design in Supplementary Fig. S2a). Body temperature and weight at the time of surgery and at the time of sacrifice did not vary between the groups (Supplementary Fig. S2b-c). There was a significantly lower neuropathological score (Fig. 1a), as judged by cresyl violet staining, in mice treated with caffeine directly after HI (0 h) (*p* = 0.0415) but not after 6 h, 12 h or 24 h, compared with the PBS-treated controls. In order to assess regional atrophy, MAP2 staining was performed in the 0-h caffeine-treated samples and in the PBS group. The tissue loss analysis revealed 25% protection in the striatum (level 1) in mice receiving caffeine compared to the vehicle-treated group (*p* = 0.0321) and no sex-specific differences were observed (Fig. 1b and Supplementary Fig. S3).

### A Single Dose of Caffeine Partially Restores Behavioural Deficits 2 Weeks After Injury

Two weeks after, HI mice were submitted to behavioural experiments for three consecutive days. The mice were placed in unfamiliar open field arenas and locomotion was videotaped for 30 min. As previously reported [21, 22], mice subjected to HI showed increased motor activity, indicative of anxiety behaviour and lack of retentiveness, which was instead normalised by early caffeine administration (0 h) to the level of non-injured animals. On the contrary, the groups receiving caffeine at later time points did not show any habituation to the arena (Fig. 1c).

Rotarod motor test was performed on the last day of behavioural evaluations and the average of 5 consecutive trials was calculated. No significant difference in the time spent on the rotating cylinder was observed between the PBS- and caffeine-treated mice at any of the time points (Fig. 1d).

Further morphological, inflammation and cellular analyses to characterise the effects of caffeine were then carried out only in the 0-h group.

### A Single Dose of Caffeine Decreases Tissue Loss and Reactive Astrogliosis

**Fig. 2:**
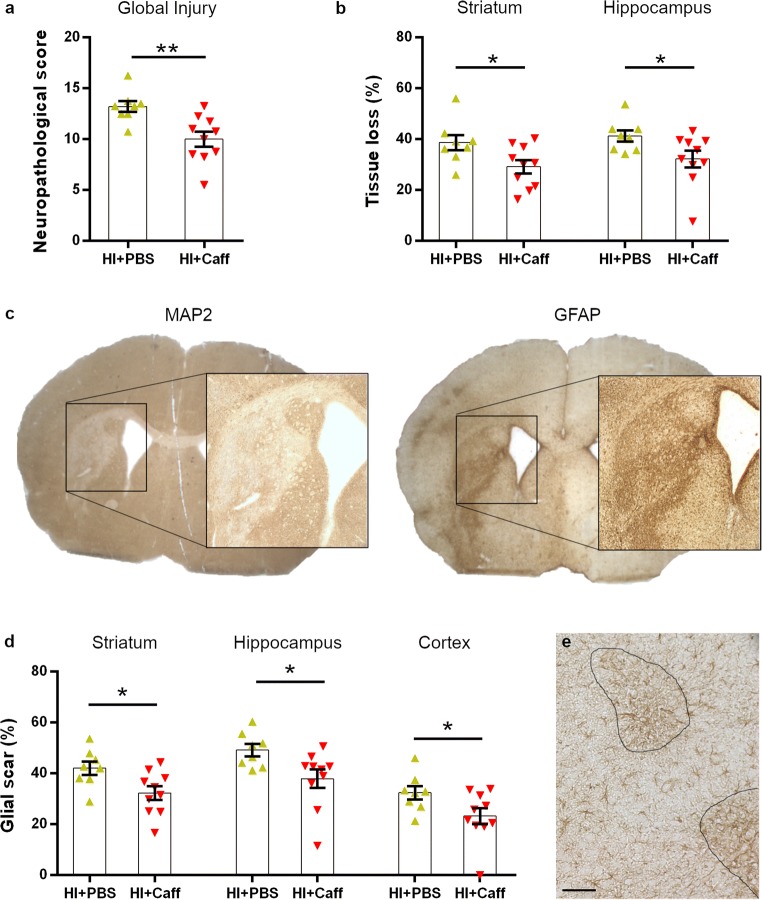
Short-term evaluation of caffeine treatment (P15). Mice were subjected to HI and received caffeine (*n* = 10) or PBS (*n* = 8) acutely after injury (0 h). Neuropathological score in sections stained with cresyl violet (**a**), tissue loss calculated by MAP2-stained tissue at the level of the striatum and hippocampus (**b**). Example of overlapping areas between the tissue loss (MAP2) and astrogliosis (GFAP) after HI injury (**c**). Glial scar percentage calculated by GFAP staining in different brain regions (**d**) and an example of reactive astrocytes contouring at × 10 with scale bar 100 μm (**e**). Data are presented as mean ± SEM with *p < 0.05 and **p < 0.01 (unpaired *t* test)

In order to investigate the impact of caffeine in the injured brain, a second set of mice was treated acutely (0 h) after HI and sacrificed 5 days after injury for further analysis (Experimental design in Supplementary Fig. S4a). Body temperature and weight at the time of surgery and at the time of sacrifice did not vary between the groups (Supplementary Fig. S4b-c). Neuropathological scoring was calculated as before on cresyl violet-stained sections to confirm the protective effect of caffeine in comparison with mice injected with PBS alone (*p* = 0.0037) (Fig. 2a). Tissue sections of consecutive section series were stained for MAP2 and GFAP to evaluate the degree of neuronal loss and astrogliosis. Compared with the PBS group, MAP2 expression revealed reduced atrophy at the striatum (level 1; *p* = 0.0306) and the hippocampus levels (level 2; *p* = 0.0446) in caffeine-treated mice (Fig. 2b and Supplementary Fig. S5). No-sex specific differences were observed.

Next, the area covered by the glial scar was analysed. A higher concentration of astrocytes with hypertrophic cell bodies (Supplementary Fig. 6), indicating a reactive phenotype, was observed in the areas affected by neuronal loss, and we thus proceeded to measure the regions where the GFAP^+^ hypertrophic cells were condensed (Fig. 2c). Similarly to the MAP2 expression analysis, the area covered by the glial scar was reduced in mice that received caffeine in the striatum (level 1; *p* = 0.0211), hippocampus (level 2; *p* = 0.0263) and in particular in the cortices (*p* = 0.0461) (Fig. 2d and Supplementary Fig. 7). Specifically, a higher concentration of GFAP^+^ cells was observed in the cortex between layers 2/3 and 5 even though minimal observable injury was detected in the same area (Fig. 2e).

### A Single Dose of Caffeine Decreases the Number of TUNEL^+^ Cells

**Fig. 3:**
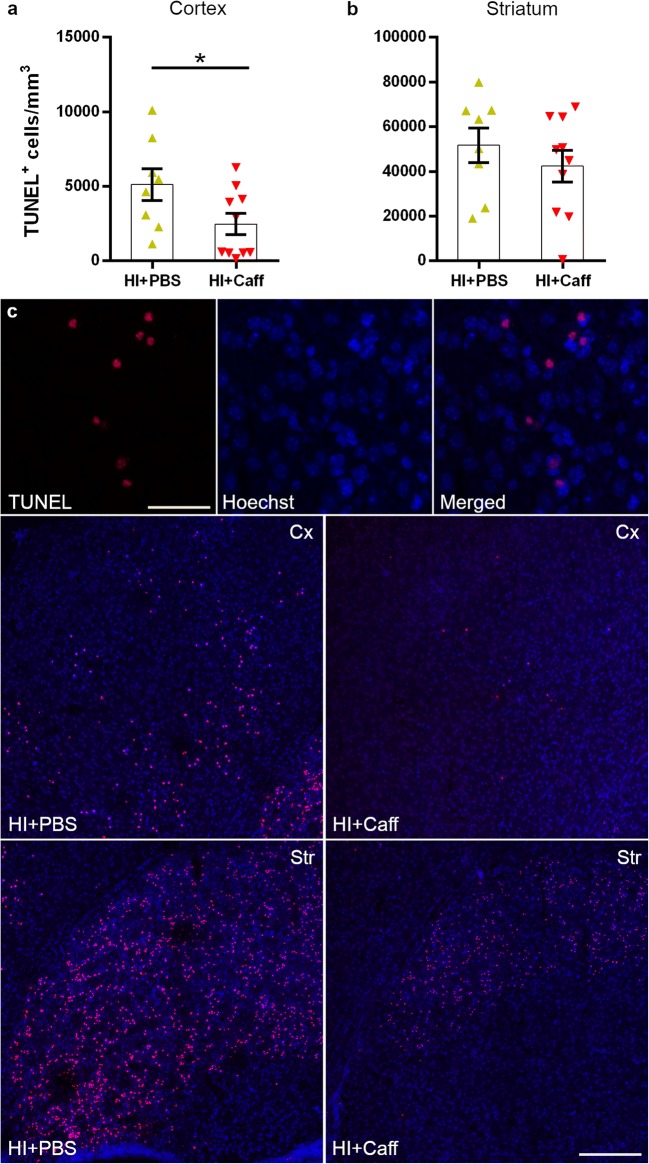
Analysis of apoptotic cells using TUNEL staining. Caffeine reduced the number of apoptotic cells in the cortex (**a**) and striatum (**b**) of mice after HI (*n* = 10) if compared to the PBS group (*n* = 8). Example of TUNEL^+^ cells at × 40 with scale bar 50 μm in the small squares and representative tiled-images of the cortex (Cx) and striatum (Str) at × 10 with scale bar 300 μm in the big squares (**c**). Data are presented as mean ± SEM with *p < 0.05 (unpaired *t* test)

Stereological analysis of TUNEL^+^ cells in the same astrogliotic area, namely the cortex and the striatum, was performed to assess the effect of caffeine. The number of apoptotic cells that incorporated TUNEL staining in their fragmented DNA was significantly reduced in the cortex of mice receiving caffeine (*p* = 0.0495), but not in the striatum, even though a similar trend was observed (Fig. 3).

### A Single Dose of Caffeine Decreases Microglia Activation

**Fig. 4:**
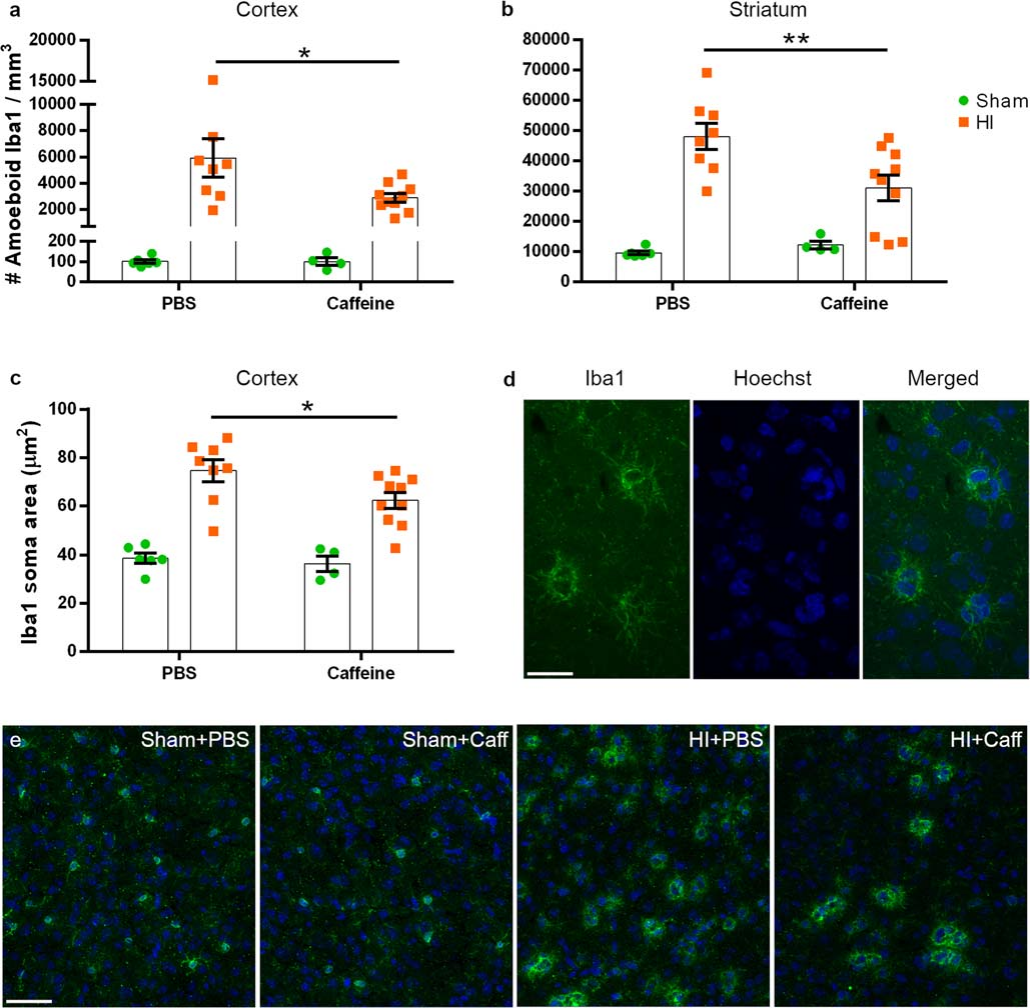
Caffeine modulates microglial cells’ density and morphology. The density of Iba1^+^ amoeboid microglia was reduced in the cortex (**a**) and striatum (**b**) of mice receiving caffeine (*n* = 10) compared to the PBS group (*n* = 8). The area of the soma of phagocytic Iba1^+^ cells was reduced after caffeine treatment in the cortex (**c**). Sham animals receiving caffeine (*n* = 4) did not show any alteration of microglia numbers or shape when compared to the Sham + PBS group (*n* = 6). Example of Iba1 staining at × 40 with scale bar 30 μm (**d**) and representative images of the cortices at × 20 with scale bar 50 μm (**e**). Data are presented as mean ± SEM with *p < 0.05 and **p < 0.01 (two-way ANOVA with Sidak’s multiple comparison test)

Iba1^+^ cell density and morphology was analysed in the areas most affected by the injury and the glial scar. The strong microglia activation caused by HI was significantly reduced in the caffeine-treated group in both the cortex (*p* = 0.0220) and the striatum specifically in the area of the caudo-putamen (*p* = 0.0048) compared to the PBS-treated mice (Fig. 4a, b). In addition, a difference in size of Iba1^+^ cells in the cortices was observed, and thus a morphological analysis of the microglia was performed. The cell size, measured by contouring the area of the soma, was significantly reduced (*p* = 0.0297) in the caffeine-treated group compared with the PBS-treated mice (Fig. 4c–e).

### A Single Dose of Caffeine Prevents Dysmyelination

**Fig. 5:**
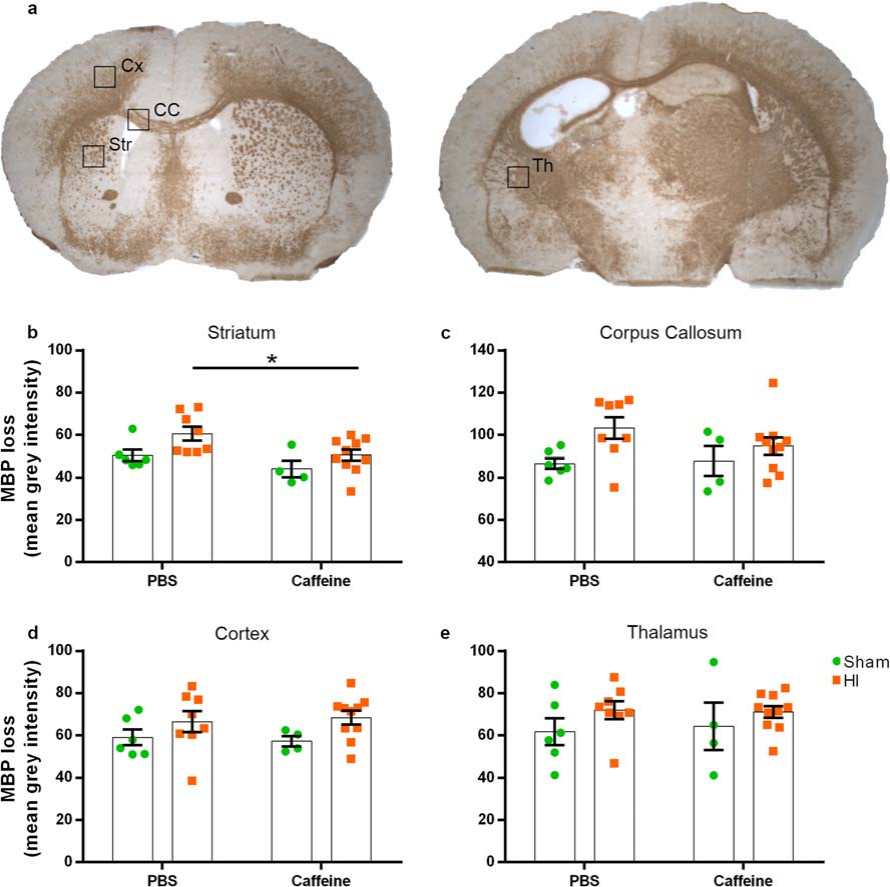
Caffeine prevents white matter injury in the striatum. Myelin basic protein (MBP) density was analysed in four different brain areas, namely the cortex (Cx), striatum (Str), corpus callosum (CC) and thalamus (Th) as indicated from the squares (**a**). HI injury led to a significant loss of myelination in almost all the analysed brain areas. Injured mice receiving caffeine (*n* = 10) showed a less-extensive MBP loss in the striatum (**b**) but not observable differences were found in the corpus callosum (**c**), cortex (**d**) or thalamus (**e**) if compared to PBS group (*n* = 8). Sham animals receiving caffeine (*n* = 4) did not show any anomaly compared to the sham mice receiving PBS (*n* = 6). Data are presented as mean ± SEM with *p < 0.05 (two-way ANOVA with Sidak’s multiple comparison test)

Even though white matter damage is less pronounced after HI at P10, MBP staining was performed to assess whether caffeine exerted a protective effect in different brain areas. White matter analysis was performed by measuring the mean grey intensity of MBP-stained tissue and normalised to the background in three × 40 images per area of interest in each animal (Fig. 5a). These data showed that HI led to loss of MBP density in mice receiving PBS. Injured animals in the caffeine group, however, showed white matter protection in the striatum compared to the PBS-treated group (*p* = 0.0302) (Fig. 5b) and a tendency to a lower MBP loss, indicative of higher myelin density, in the corpus callosum although not statistically relevant (Fig. 5c). No differences between treatments were observed in the cortices and thalamus (Fig. 5d, e).

### A Single Dose of Caffeine Is Associated with Altered Inflammation-Related Gene Expression

**Fig. 6:**
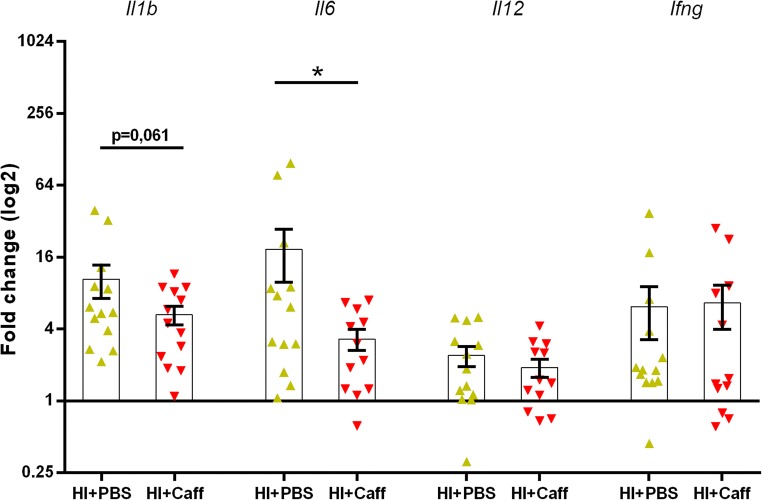
Caffeine prevents upregulation of the expression of pro-inflammatory genes 24 h after HI. RT-qPCR analysis of *Il1b*, *Il6*, *Il12* and *Ifng* genes in brain homogenates derived from ipsilateral cortices of HI mice receiving either caffeine (*n* = 13) or PBS (*n* = 12) normalised to control animals. The expression of each gene was calculated by normalisation to the average of housekeeping genes *B-actin* and *Rpl13a*. Data are presented as mean ± SEM with *p < 0.05 (unpaired *t* test)

To investigate whether caffeine-treatment influenced the inflammatory cascade, cortices from the ipsilateral hemispheres were dissected, and RT-qPCR was performed on homogenised samples recovered 24 h after HI. The expression of four genes of interest, normalised to internal controls, showed a strong trend in the expected direction for *Il1b* (*p* = 0.0608) and a significant downregulation for *Il6* (*p* = 0.0340). No differences were seen in the expression of *Il12* and *Ifng* (Fig. 6).

## Discussion

Caffeine is commonly used in the clinic due to its wide therapeutic index, its rapid distribution in the brain and a long half-life in infants compared to adults [23–25]. There are several studies evaluating the short- to long-term neuroprotective effects of caffeine, but there is little evidence describing the therapeutic time window of caffeine after HI in term-neonates.

In the present study, we evaluated caffeine administration at different time points and identified its post-injury effectiveness in neonatal mice. The major finding was that caffeine reduced moderate-severe brain damage only if given directly after HI, but not when administered at later time points. Both grey and white matter were protected by caffeine treatment, and the number of amoeboid microglia, apoptotic cells, the area of astrogliosis and the expression of pro-inflammatory cytokines were decreased compared to controls.

We previously reported neuroprotection and improved rotarod and open-field performance after caffeine in a mild form of HI [10]. Other studies have confirmed behavioural ameliorations in rodents subjected to injury in the perinatal period after higher doses of caffeine or multiple administrations [12, 26]. Herein, we demonstrated that an acute caffeine treatment reduced the extent of brain damage also in a moderate-to-severe injury model and led to a partial functional recovery, as shown by open-field behavioural experiments. This confirms the neuroprotective effects of caffeine reported in humans [9].

It has been shown that the binding of adenosine to A2a receptors during ischaemia may lead to cell death [27, 28]. Our current findings thus suggest that an imminent blockage of the ARs is needed to maintain the beneficial effects seen in the acute treatment and that caffeine’s antagonistic effect is limited at later time points.

Indeed, when administered acutely, a single dose of 5 mg/kg caffeine reduced MAP2 tissue loss in the hippocampus and striatum, regions with the higher expression of the A1R and A2aR respectively. This suggests a neuroprotective mechanism that involves both receptors [28].

Similarly, the area covered by reactive astrocytes was reduced at the level of the striatum and hippocampus in the caffeine-treated group when compared with the control group 5 days after HI. Mice receiving caffeine also presented less extensive glial scars in the cortices, even though no comparable MAP2 injury was observed. This was seen specifically between layers 2/3 and less prominently, in layer 5. The reactivity of astrocytes in these areas was however associated with an elevated number of apoptotic cells as judged by TUNEL staining. In accordance with previous findings [11], the presence of TUNEL^+^ cells in caffeine-treated mice was significantly reduced and a smaller glial scar was consequently observable.

Studies have shown that astrogliosis is protective by confining the effect of the injury and that this is essential to provide factors for cell survival [29, 30]. Under pathological conditions, though, hyper-reactive astrocytes exacerbate the injury and cause both neurotoxicity and inflammation, and therefore increase the level of pro-inflammatory factors [31, 32]. Caffeine was previously shown to reduce astrocyte immunoreactivity and proliferation by selectively antagonizing A2aR [33], thus suggesting a direct modulatory mechanism in the inhibition of the glial scar formation in the caffeine-treated group.

In accordance with the smaller lesion and a reduced cell death in mice receiving caffeine, we also observed decreased microglial activation as shown by the diminished soma size and a smaller amoeboid cell density in the cortex and striatum. Even though this reduced inflammation could be the result of a contained lesion due to the caffeine treatment, a direct caffeine-induced modulation in microglia has been suggested. Recent findings have demonstrated that adenosine agonists in microglia culture lead to an increase of M1 marker expression [34], and moreover, activation of the A2aR in microglia was associated with cytokine release and process retraction in vivo suggestive of an activation state [35, 36]. In contrast, caffeine suppresses the generation of pro-inflammatory mediators [37], and the blockade of A2aR was indeed reported to reduce microglial activation both in vivo and in vitro [38], thus confirming the present findings.

Lastly, we have shown that caffeine can preserve white matter development, specifically in the striatum, where a higher concentration of MBP^+^ fibres are detected, and others have also reported an enhancement of myelination in caffeine-treated mice in a model of periventricular white matter injury [39]. Caffeine indeed seems also to prevent the hypoxia-induced demyelination by reversing the altered maturation of oligodendrocytes progenitors and promote their normal development via inhibiting the A1R [40–42].

Taken together, these results suggest that caffeine has a great potential as therapeutic agent to treat HI. Indeed, a single administration of 5 mg/kg could reduce loss of gross brain volume and myelinated fibres, decrease glial activation and ameliorate behavioural outcomes. A higher dose of caffeine or multiple injections might be needed to further rescue the cellular reorganization and the plasticity effects that lead to functional impairment in a moderate-to-severe lesion.

## Conclusion

The present findings indicate that a single dose of caffeine given acutely after HI leads to neuroprotection, immunomodulation and partial functional recovery. Our data indeed show improvement in open-field behavioural tests, reduced grey and white matter loss and apoptotic cell density, decreased amoeboid microglia and area of astrogliosis and modulation of the expression of pro-inflammatory cytokines in mice treated with caffeine compared to controls.

Based on our results, the time window for caffeine therapeutic effects was very short. Further studies are needed to confirm the dose and number of administrations required for caffeine to restore further the functional impairment in moderate-to-severe HI lesions.

## Electronic supplementary material

ESM 1 (DOCX 9342 kb)
