# Supplementary material for: Defining a Time Window for Neuroprotection and Glia Modulation by Caffeine After Neonatal Hypoxia-Ischaemia
Source: Mol Neurobiol. 2020 Jan 23;57(5):2194–205. doi: 10.1007/s12035-020-01867-9 (PMC7170835; doi:10.1007/s12035-020-01867-9)
Supplement: Supplementary file 1 — (DOCX 9342 kb) [file 12035_2020_1867_MOESM1_ESM.docx]

**SUPPLEMETARY INFORMATION**

**Title**

Defining a time window for neuroprotection and glia modulation by caffeine after neonatal hypoxia-ischaemia

**Authors**

Elena Di Martino^a,#^, Erica Bocchetta^a^, Shunichiro Tsuji^a,b^, Takeo Mukai^a^, Robert A. Harris^c^, Klas Blomgren^a,d^ and Ulrika Ådén^a^

**Affiliation**

^a^ Dept. of Women’s and Children’s Health, Karolinska Institutet, Sweden

^b^ Dept. of Obstetrics and Gynaecology, Shiga University of Medical Science, Japan

^c^ Dept. of Clinical Neuroscience, Karolinska Institutet, Centre for Molecular Medicine, Karolinska Hospital, Solna, Sweden

^d^ Dept. of Paediatric Oncology, Karolinska University Hospital, Stockholm, Sweden

^#^ Corresponding author: [elena](mailto:elena).di.martino@ki.se

Dept. of Women’s and Children’s Health, Karolinska Institutet

BioClinicum J9:30, Akademiska Stråket 1, 17164 Stockholm, Sweden


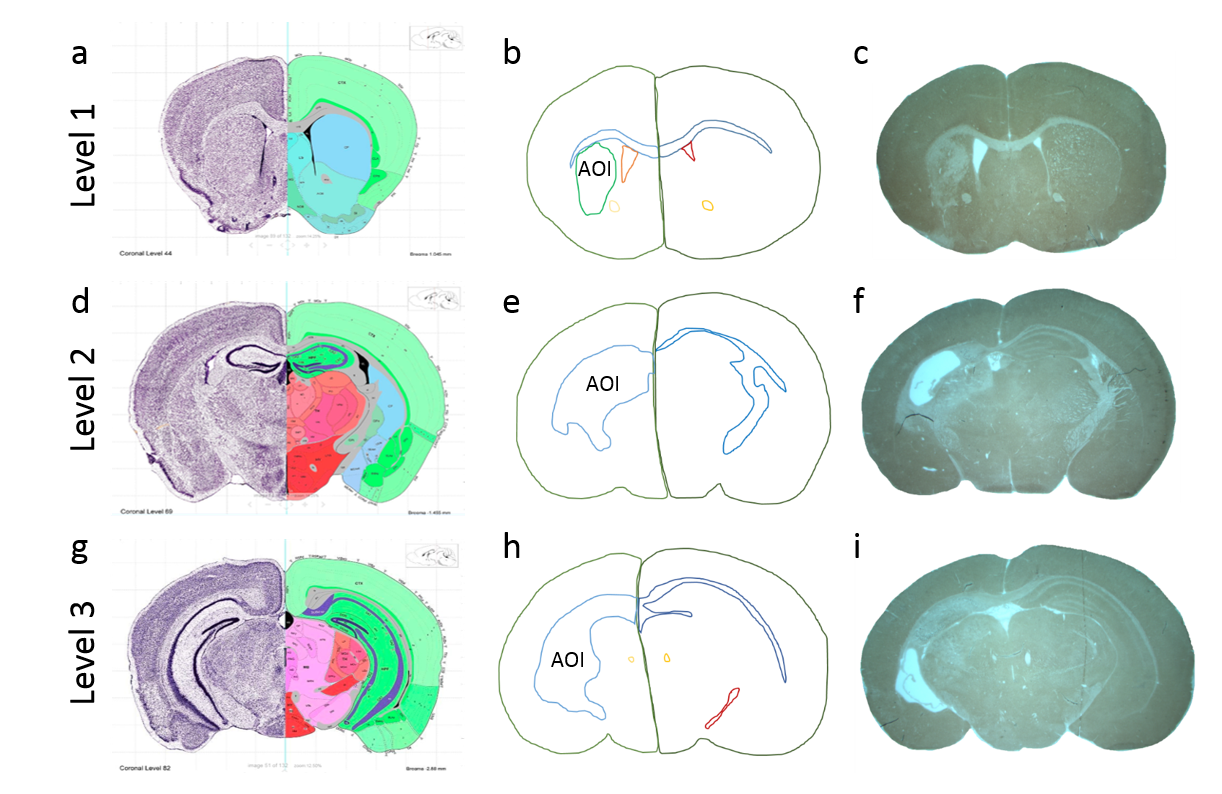


**Supplementary Fig. S1** Levels of interest as represented by Allen Reference Atlas (a,d,g), contouring strategy (b,e,h) with delineated the area of interest (AOI) and other MAP2 unstained brain areas on an example of MAP2 staining (c,f,i).


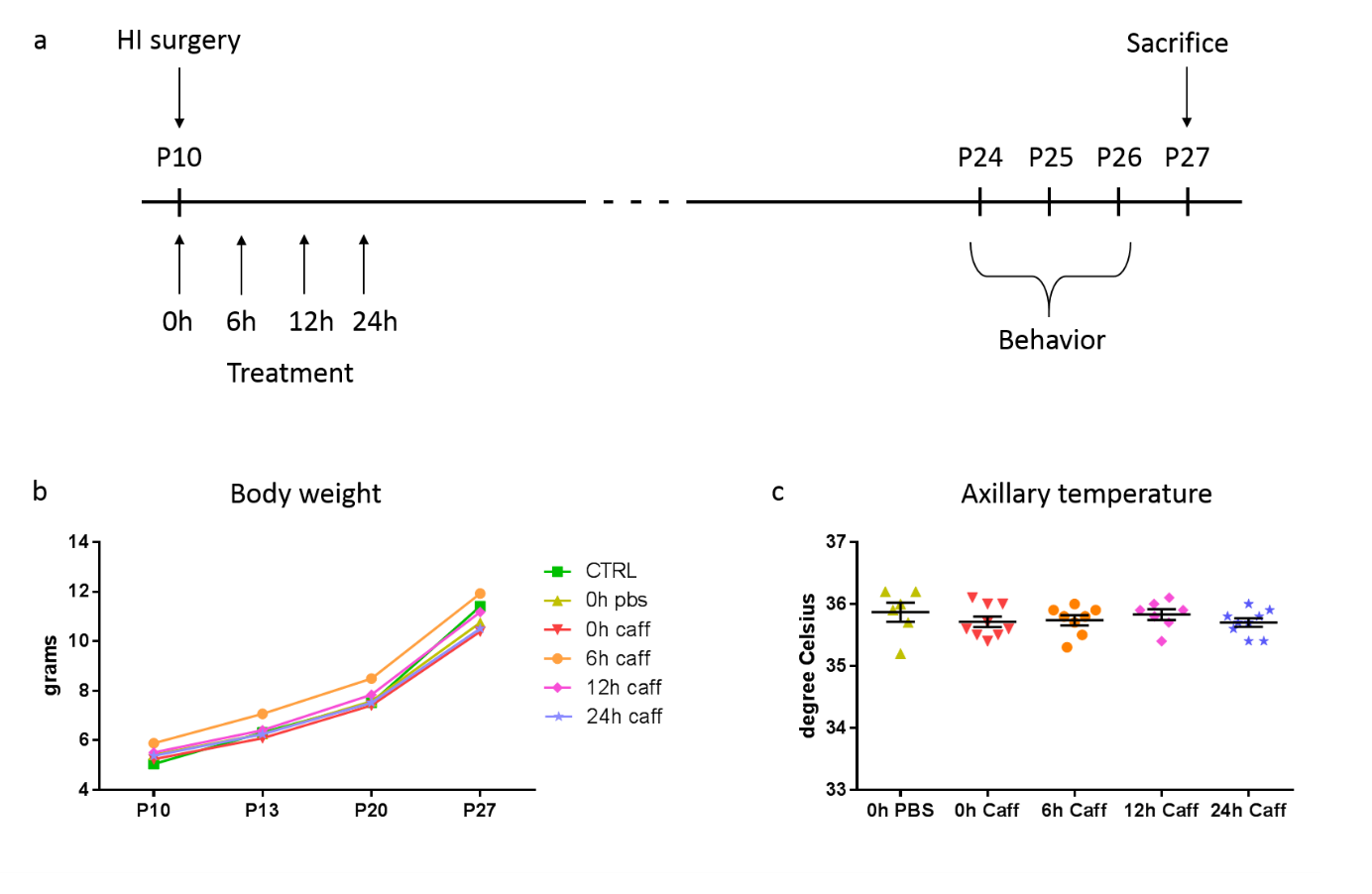


**Supplementary Fig. S2** Study design of the long-term experiment (a) and general parameters for surgical procedure: no difference between groups was observed in the body weight over time (b) and axillary temperature after injury (c). Data are presented as mean ± SEM. One-way ANOVA with Bonferroni’s multiple comparison test, p>0.05.

**
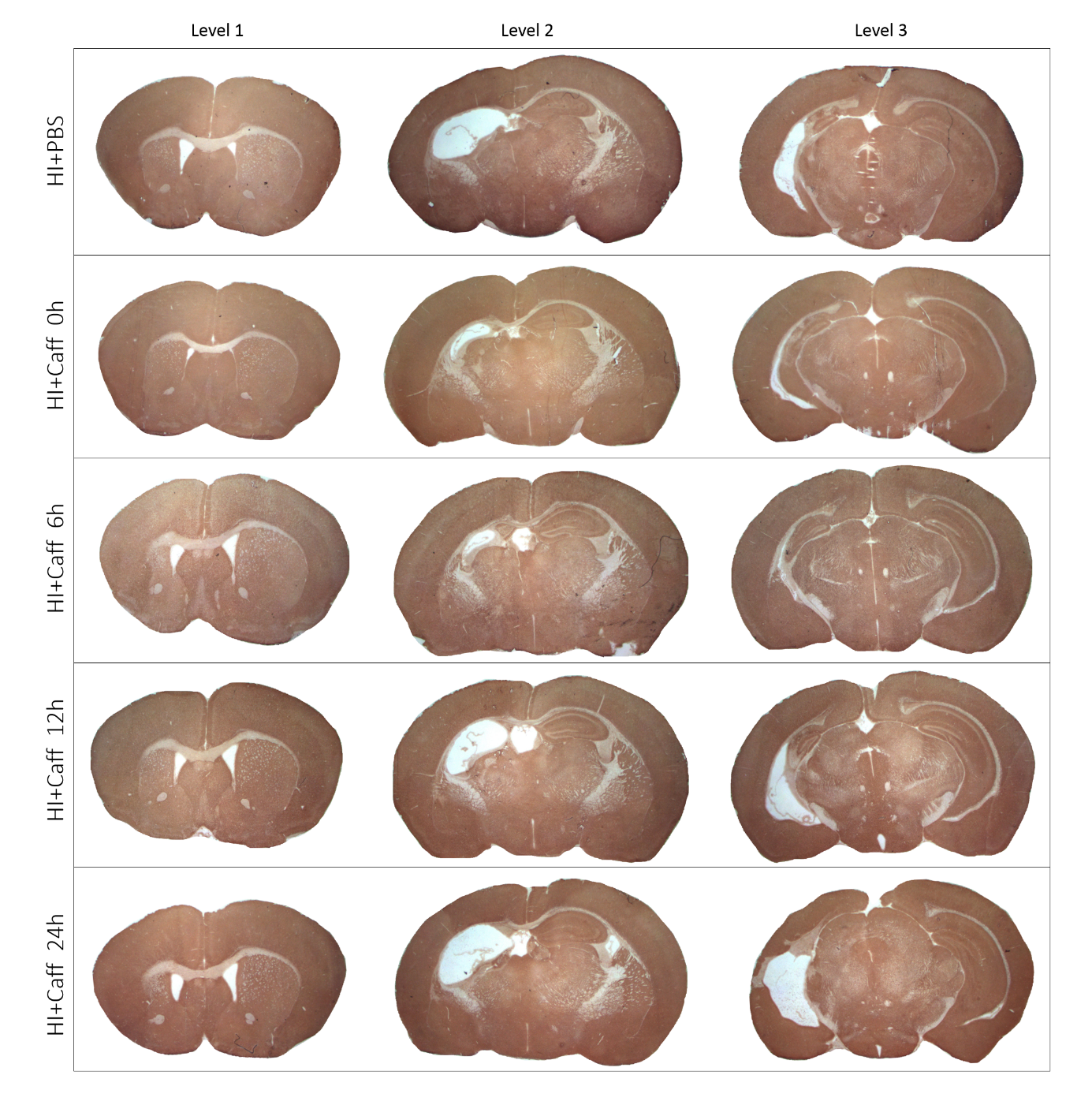
**

**Supplementary Fig. S3** Representative MAP2 images of all groups and levels of interest

**
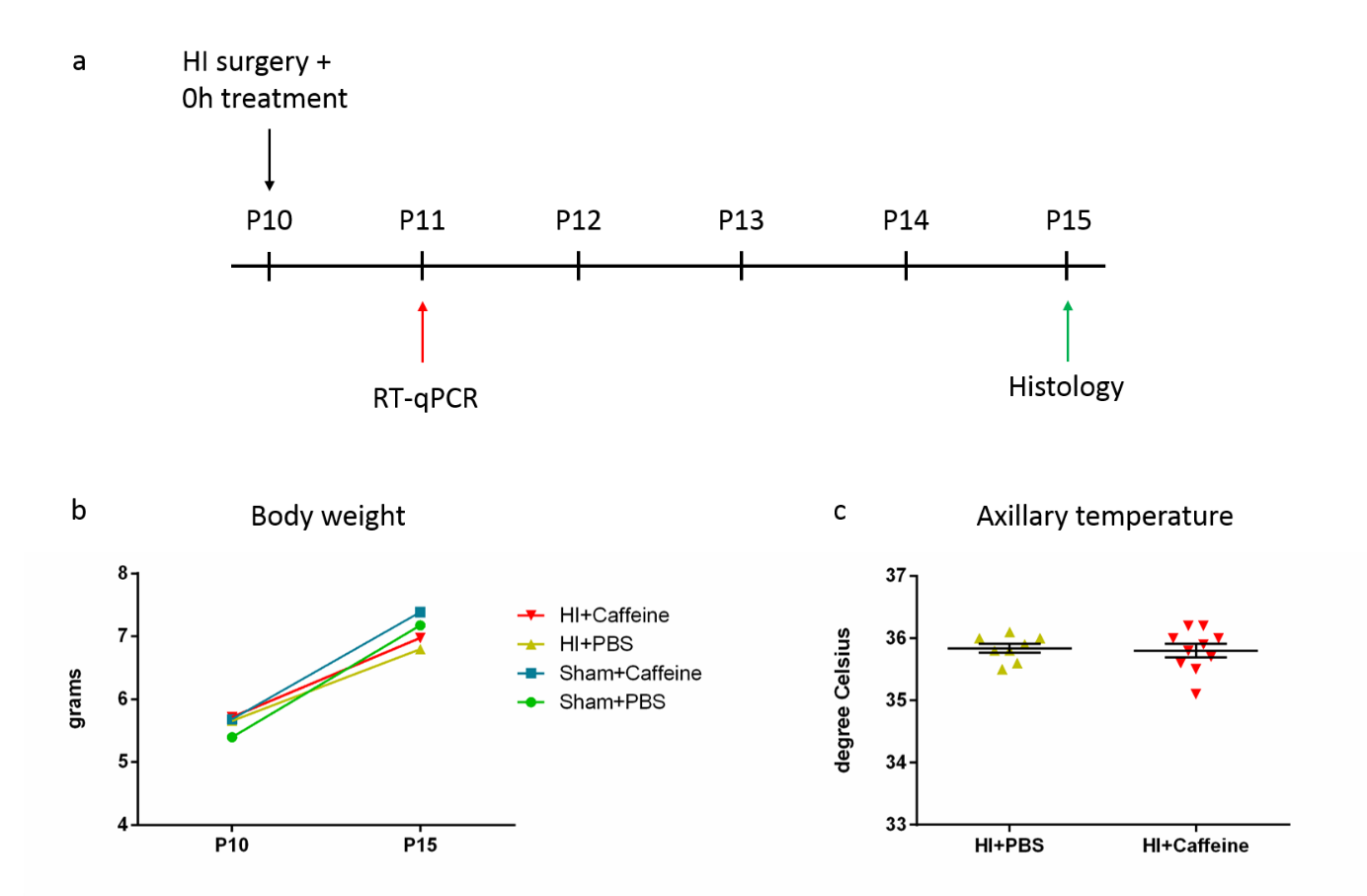
**

**Supplementary figure S4**: Study design of the short-term experiment (a) and general parameters for surgical procedure: no difference between groups was observed in the body weight over time (b) and axillary temperature after injury (c). Data are presented as mean ± SEM. T-test with p>0.05.

**
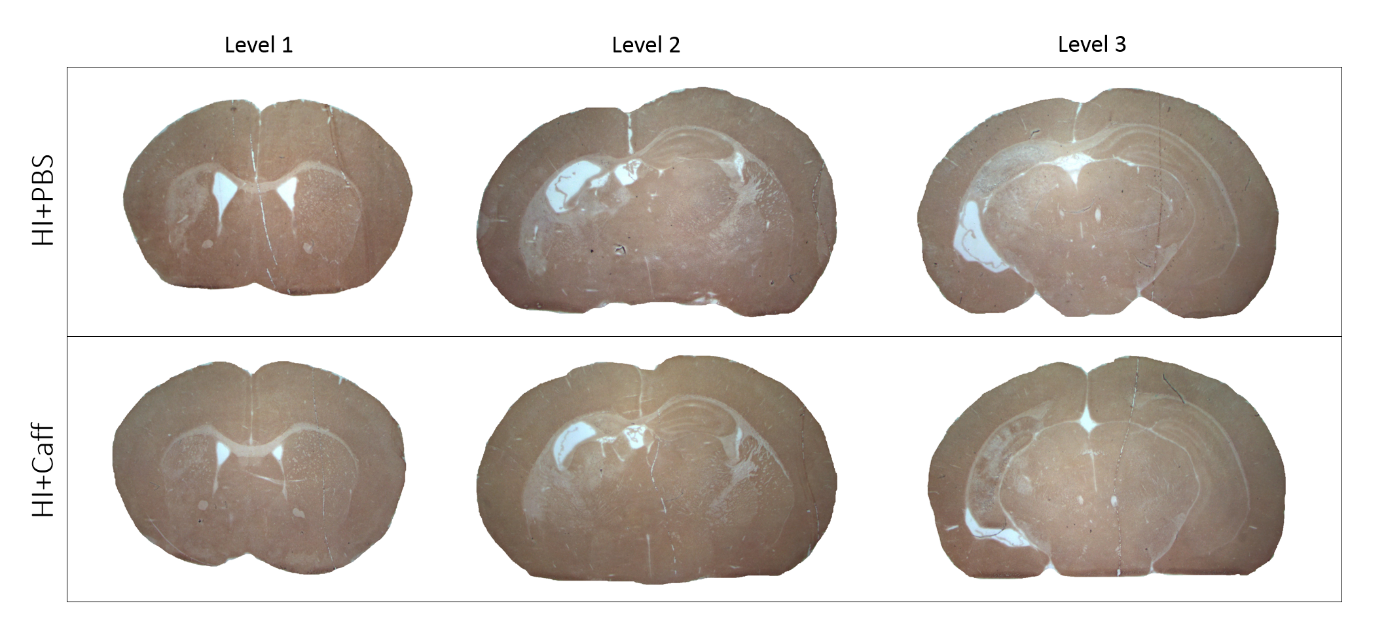
**

**Supplementary Fig. S5** Representative MAP2 images of the injured groups in all levels of interest.

**
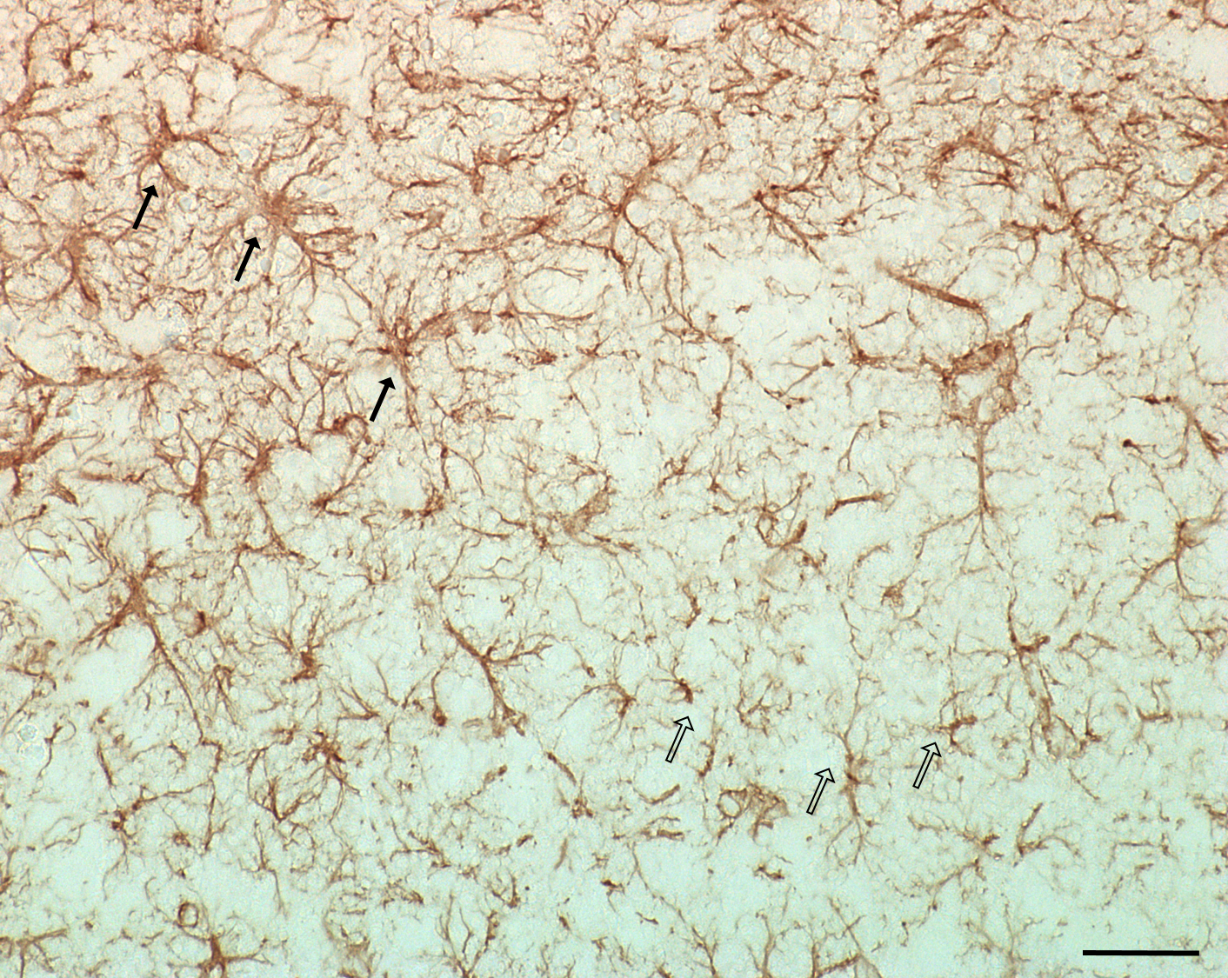
**

**Supplementary Fig. S6** Representative image of GFAP^+^ cells at 40X: astrocytes with hypertrophic bodies and processes in the upper part of the image (full arrows) and normal astrocytes in the lower part of the image (empty arrows). Scale bar 50 μm.


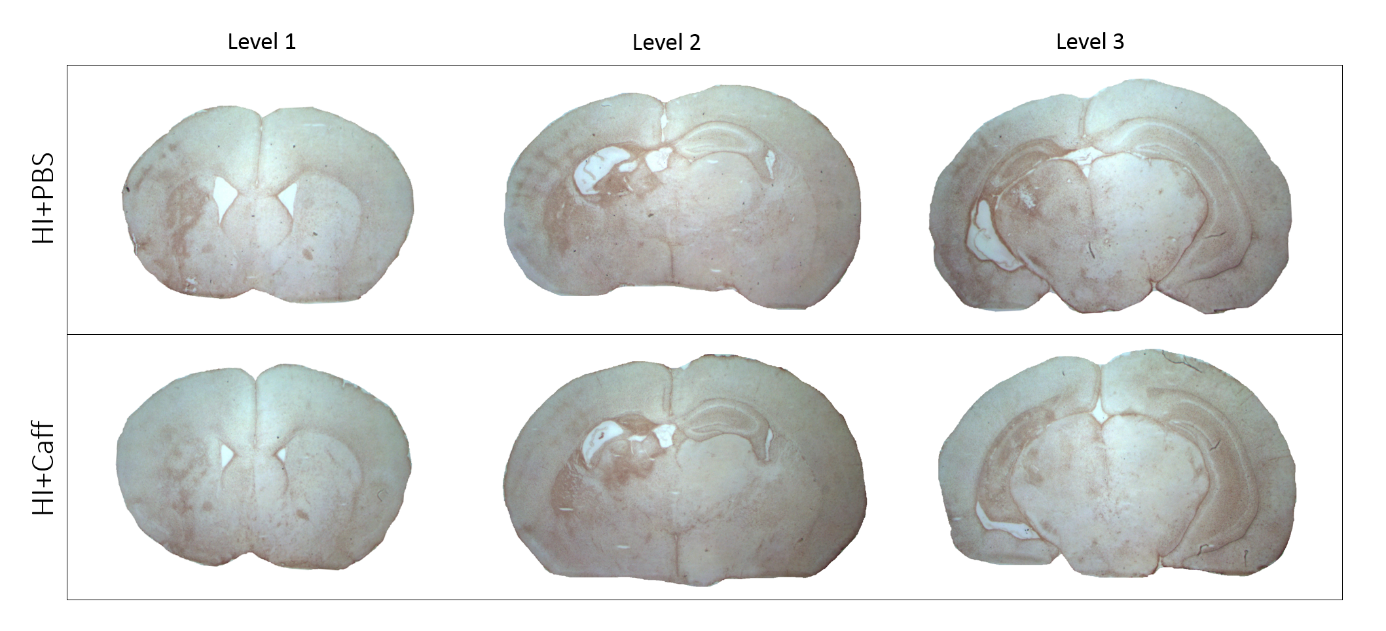


**Supplementary Fig. S7** Representative GFAP images of the injured groups in all levels of interest.

**Supplementary Table 1**

| *Rpl13a* | Forward | 5’-TGACAAGAAAAAGCGGATGGTG-3’ |
| --- | --- | --- |
|  | Reverse | 5’-GCTGTCACTGCCTGGTACTT-3’ |
| *Il1b* | Forward | 5’-TGCCACCTTTTGACAGTGATG-3’ |
|  | Reverse | 5’-TGATGTGCTGCTGCGAGATT-3’ |
| *B-actin* | Forward | 5’-AGATCAAGATCATTGCTCCTCCT-3’ |
|  | Reverse | 5’-ACGCAGCTCAGTAACAGTCC-3’ |
| *Il12* | Forward | 5’-AAATGAAGCTCTGCATCCTGC-3’ |
|  | Reverse | 5’-TCACCCTGTTGATGGTCACG-3’ |
| *Ifng* | Forward | 5’-ACGGCACAGTCATTGAAAGCCTAGA-3’ |
|  | Reverse | 5’-TGTCACCATCCTTTTGCCAGTTCC-3’ |
| *Il6* | QT00098875 | Mm_IL6_1_SG (Qiagen) |
